# Effects of Acute and Moderate Caffeine Doses on Sport Climbing Performance: A Randomized Controlled Trial

**DOI:** 10.3390/nu18020284

**Published:** 2026-01-16

**Authors:** Alejandra Ruiz-López, Juan Jesús Montalvo-Alonso, Iván Martín-Rivas, Marta del Val-Manzano, Carmen Ferragut, David Valadés, Marta Barrios-Egea, Paola Gonzalo-Encabo, Alberto Pérez-López

**Affiliations:** Departmento de Ciencias Biomédicas, Área de Educación Física y Deportiva, Faculty of Medicine and Health Sciences, University of Alcalá, 28801 Madrid, Spain

**Keywords:** sport climbing, grip strength, pull up strength, caffeine, sports performance

## Abstract

**Background/Objectives**: Caffeine is a well-established ergogenic aid in many strength- and endurance-based sports, but its efficacy in sport climbing remains underexplored despite the sport’s unique physical demands on grip strength, power, and muscular endurance. Therefore, this study examined the acute impact of a low caffeine dose (3 mg/kg) on climbing-specific performance, including pull-up and grip tests, in intermediate-advanced climbers. **Methods**: In a triple-blind, randomized, crossover design, thirteen male climbers (age: 28.2 ± 8.6 years) completed two experimental trials (caffeine vs. placebo). Performance was assessed via a pull-up one-repetition maximum (1RM) and power test at various loads, a pull-up muscular endurance test, and grip tests including maximum dead-hang time, maximum dead-hang strength, and rate of force development (RFD). **Results**: Caffeine did not significantly enhance performance in any measured variable. While a non-significant increase in peak power was observed at 80% 1RM (+8.0%, 95% CI: −0.232 to 0.304, *p* > 0.05, g = 0.348), effects at other loads and on pull-up endurance were trivial based on effect size (e.g., repetitions: +3.3%, 95% CI: −3.30 to 4.37, *p* = 0.292, g = 0.061). For grip metrics, caffeine was associated with a modest reduction in endurance time (+7.4%, *p* = 0.162, g = 0.171) and a slight increase in maximum strength (+2.4%, *p* = 0.060, g = 0.120). RFD was unaffected (*p* > 0.169, g < 0.13). Despite the lack of objective improvement, participants reported significantly greater subjective feelings of strength, energy, and alertness with caffeine (*p* < 0.05). **Conclusions**: A 3 mg/kg dose of caffeine, while altering psycho-physiological state, did not elicit statistically or practically meaningful ergogenic effects on pull-up or grip performance in climbers. Higher doses or sport-specific performance tests should be investigated in future research.

## 1. Introduction

In recent years, climbing has experienced remarkable growth, fueled by its inclusion in the Olympic Games, increased media exposure, and the rapid expansion of indoor climbing facilities [1]. This surge in popularity has attracted both recreational participants and professional athletes, who are all seeking to optimize their performance in a sport that presents complex physical and psychological challenges [2].

Climbers must manage a combination of nutritional, physiological, and psychological demands [3,4]. From a physiological perspective, maintaining a high power-to-mass ratio, delaying muscular fatigue, promoting efficient recovery, and specially achieving high grip strength capacity are crucial for optimal performance [5,6]. Despite these demands, research specifically addressing nutritional strategies and supplementation to enhance climbers’ performance remains limited, with few comprehensive reviews available [7,8]. Among available supplements, caffeine stands out as one of the most widely studied and frequently consumed ergogenic aids across different sports and exercise modalities. Evidence indicates that caffeine doses between 3 and 6 mg/kg are sufficient to enhance performance, while the risk of adverse side effects increases with higher doses [9,10]. Lower doses (≈3 mg/kg) are associated with minimal side effects, whereas doses approaching or exceeding 6 mg/kg increase the likelihood of negative symptoms, including sleep disturbances, which may impair recovery, an important consideration for climbers who often train in the afternoon or evening.

Caffeine acts primarily as an adenosine receptor antagonist [11], increasing neurotransmitter release and reducing perceived exertion and fatigue, key factors in high-intensity intermittent sports such as climbing. In addition, caffeine has been shown to improve muscle strength, power, and endurance [12,13] by increasing motor unit activation [14] and enhancing intracellular calcium release in muscle fibers [15], thereby supporting force production during repeated or sustained efforts. Collectively, this may lead to the assumption that caffeine could enhance climbing performance. However, only one published study has directly examined the effects of caffeine intake on climbing performance, employing an energy drink with multiple active ingredients and focusing on grip strength outcomes [16].

Surveys of supplement use among climbers report mixed findings. Some suggest that caffeine is among the least commonly used aids [4,17], while others note that it is the most frequently consumed, especially by advanced athletes [18]. Reported motivations for use often draw from broader sports science literature, including reduced pain perception, faster recovery, and improved focus. Moreover, studies in non-climbing contexts indicate that caffeine can positively influence grip strength and endurance [19], pull-up strength [20], muscular endurance [12] and rate of force development [21], with these factors being highly relevant to climbing performance. Given these gaps, the present study aimed to examine the acute effects of low-dose caffeine ingestion (3 mg/kg) on key climbing performance variables, including maximal and endurance pull-up strength, maximal and endurance finger flexor strength during dead-hang tests, and rate of force development.

## 2. Materials and Methods

### 2.1. Participants

Sixteen male climbers were initially recruited for this study, of whom thirteen completed all assessments. They had a mean age of 28.2 ± 8.6 years, a climbing experience of 6.3 ± 4.3 years, at an intermediate-advanced level following Draper et al. [22], and were low-to-moderate caffeine consumers (1.21 ± 1.12 mg/kg/day) according to Filip et al. [23] classification. Participant characteristics are summarized in Table 1. Inclusion criteria were as follows: (a) healthy individuals without physical limitations preventing completion of the protocol; (b) age ≥ 18 years; (c) a minimum of three months of continuous climbing practice; (d) ability to hang for at least 5 s from an 18 mm edge, to ensure that participants are experienced climbers; (e) and ability to perform at least one strict pull-up with body mass.

All participants were fully informed of the experimental procedures and potential risks before providing written informed consent. The study was conducted in accordance with the Declaration of Helsinki and approved by the University Research Ethics Committee (CEIP/2024/5/117).

### 2.2. Experimental Design

**Figure 1:**
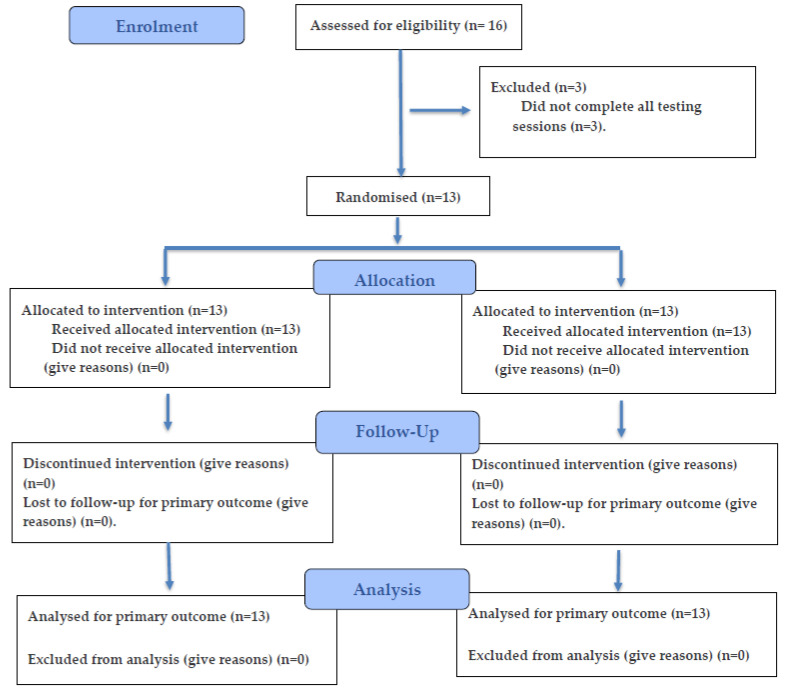
Flow diagram of the progress through the phases of a randomized trial of two groups.

A triple-blind, randomized, placebo-controlled design was used (Figure 1). Participants completed three sessions. The first session was a familiarization session in which participants performed the same test and procedures as those used in the experimental trials. Thus, the second and third sessions followed the same protocol but after ingestion of either caffeine or placebo. Visits were separated by at least 48 h to ensure adequate recovery and scheduled within a one-week window to minimize potential confounding effects from changes in sleep, nutritional, or physical activity habits. To avoid circadian effects, all testing sessions were conducted at the same time of day (≈6:30 h p.m. ± 30 min).

The treatment order was determined using a computerized block randomization (block size = 2) to ensure balanced allocation (caffeine vs. placebo order) within this small-sample study, minimizing the risk of unequal group assignments. Randomization codes were generated and held by an independent researcher not involved in data collection or analysis. All participants, investigators, and analysts remained blinded until data analysis was complete.

### 2.3. Experimental Protocol

**Figure 2:**
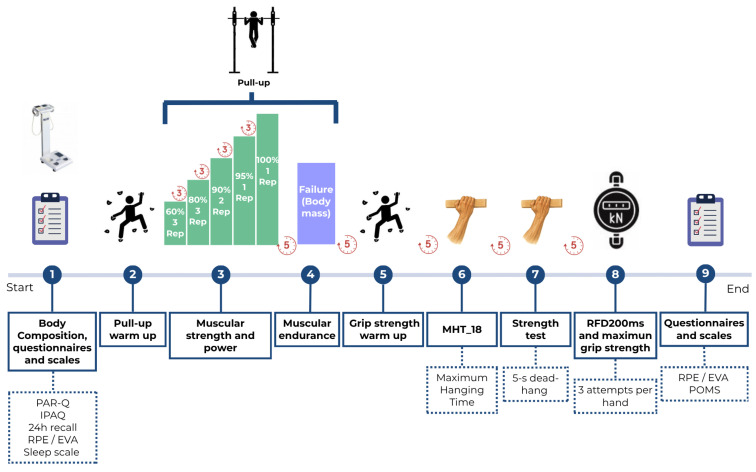
Test sequence during each testing session.

The experimental sequence is shown in Figure 2. Following the completion of body composition assessment, questionnaire administration, and a standardized general warm-up, participants undertook two pull-up performance tests in the following order: (a) pull-up muscular strength and power and (b) pull-up muscular endurance. Subsequently, after a specific warm-up targeting the finger flexor musculature, participants completed three grip performance assessments: (a) grip endurance during a dead-hang, (b) grip strength during a dead-hang, and (c) grip rate of force development (RFD). A five-minute passive rest was provided between performance tests [24]. Participants were permitted to use magnesium, and holds were brushed regularly to maintain consistency.

#### 2.3.1. Body Composition, Dietary, and Physical Activity Habits

Body composition, dietary intake, and physical activity habits were recorded on each visit. Body composition was assessed using bioelectrical impedance (Tanita MC-780MA, Tanita Corp., Tokyo, Japan). Participants recorded all foods consumed in the 24 h prior to testing, which were analyzed using the Spanish Food Composition Database (BEDCA) and Higher Education Center for Nutrition and Dietetics (CESNID) food composition tables. Participants were instructed to replicate meals across sessions and to avoid caffeine or other stimulants. Physical activity was assessed with the International Physical Activity Questionnaire (IPAQ).

#### 2.3.2. Supplementation Protocol

Participants ingested either caffeine (3 mg/kg; HSN, Granada, Spain) or placebo (3 mg/kg maltodextrin) 45 min before the testing session. This dose, chosen to limit the occurrence of side effects while remaining effective, is roughly equivalent to the caffeine content of 2–3 cups of coffee or tea, or 1–2 energy drinks. Supplements were diluted in 150 mL of flavored water (MyProtein, Northwich, UK; calorie-free) to mask the taste and smell and served in opaque shaker bottles to ensure blinding.

#### 2.3.3. Warm-Up

Participants completed two supervised and standardized warm-up routines adapted from established protocols [6,25]. The pull-up warm-up included general joint mobility, elastic-band exercises targeting major upper-body muscles, progressive climbing, and preparatory sets of scapular and full pull-ups. The grip strength warm-up consisted of progressive climbing on increasingly smaller holds, followed by three 15 s dead-hangs of increasing intensity on edges down to 18 mm. These routines last 15 min and ensure appropriate preparation and consistency across testing sessions.

#### 2.3.4. Pull-Up Test

*Pull-up one-repetition maximum and muscular strength power test*

The pull-up one-repetition maximum (1RM) was determined as the maximum total load lifted (including body mass) during the pull-up exercise, using the mean concentric velocity, and recorded using a linear transducer (Encoder, Chronojump Boscosystem, Barcelona, Spain). [26]. On this visit, the load progression was determined based on the mean concentric-phase velocity recorded in each set. During visit 1 (familiarization), the external load was increased by 5–10 kg when the mean velocity was greater than 0.30 m/s, and by 1–2 kg when it was lower than 0.30 m/s, to progressively adjust the intensity until reaching the one-repetition maximum. During visits 2 and 3 (experimental trials), the pull-up 1RM was determined by increasing loads: 3 repetitions at 60% and 80% 1RM, 2 repetitions at 90% 1RM, and 1 repetition at 95% and 100% 1RM. A 3 min passive recovery period was allowed between sets. For all repetitions, mean and peak velocity (V_mean_ and V_peak_) and power (W_mean_ and W_peak_) were recorded.

During all visits, added weights were attached to the harness waist belt using the same cord and carabiner. The encoder was placed on the floor, carefully aligned with the participants’ vertical axis, as previously reported [26], and connected to the back of the harness. A researcher was responsible for ensuring that the movement during the exercise followed a strictly linear trajectory. Additionally, the range of motion was continuously monitored, and only repetition with <5% variation between familiarization and trials were accepted.

Moreover, the same pull-up bar was used across all testing days (SLIM2 SPEKTR Pull-Up Bar, Indigo Sports, Alicante, Spain). It was securely mounted to the wall at a height sufficient to prevent participants from touching the floor during the excentric phase. The bar measured 28 mm in diameter, as recommended in previous research [26], and was 1 m in length, allowing for an appropriate grip width for each participant. Each repetition was required to be performed without swinging, leg momentum, or deviations from the proper descending position, and was held isometrically for 2 s. Grip width was measured and maintained consistently throughout all sessions. Each attempt was executed from a fully extended arm position to the chin passing above the bar.

*Pull-up muscular* *endurance test*

After a five-minute rest, using the same procedure and instruments described in the muscular strength power test, pull-up muscular endurance was evaluated. Participants performed a single set of pull-ups to task failure using their body mass. They maintained the technique described above and did not let go of the bar or rest for more than 5 s while hanging. The total number of repetitions, V_mean_, V_peak_, W_mean,_ and W_peak_ were recorded (Encoder, Chronojump Boscosystem, Spain).

#### 2.3.5. Grip Test

The following considerations were taken during the grip tests. (a) Edge depth: An 18 mm wooden edge was used to accommodate the varied skill levels and ensure safety [27]. (b) Grip type: Halfcrimp (pure or open) or slope grip were allowed; full crimp was prohibited due to joint stress [5,28]. Participants used the same grip type for all tests. (c) Dead-hang technique: Arms remained extended with scapulae retracted, avoiding swinging or neck movements to ensure standardized posture [29].

*Grip endurance during dead-hang*

After grip warm-up, participants performed the maximum hanging time (MHT) test at an 18 mm edge depth with each hand [5]. The test ended if the participant touched the ground, bent their arms, or experienced pain. Only one measurement was performed for this test on each hand [25].

*Maximum grip strength during dead-hang*

Maximum grip strength in the dead-hang position was assessed using the Strength Test (ST), which measures the maximal external load a participant can sustain for 5 s while hanging from an 18 mm edge [6,27]. During visit 1 (familiarization), the minimum number of attempts were used to determine each participant’s maximal load, and the external load progressively increased, with 3 min rest intervals between attempts. During visits 2 and 3 (experimental trials), participants performed approach sets at 25%, 50%, 75%, and 90% of their previously determined maximal load, followed by attempts at 100%. If a participant successfully completed the 100% trial, the load was progressively increased until failure, to accurately determine the maximal load of the trial.

*Grip rate of force development (RFD)*

The grip rate of force development (RFD) was determined using a force sensor (Chronojump Boscosystem, Spain; sampling frequency 160 Hz) and measuring the maximal one-hand pull (voluntary contraction) at an 18 mm edge. This instrument has been used in previous climbing studies [30,31]. The device was calibrated prior each test following manufacturer recommendations. For the extraction of maximum grip force and RFD at 200 ms values, the software’s modeled function was utilized. Data analysis involved manually selecting the initial point at which the force exceeded a 7 N threshold, after the subject had maintained a stable force of 0 N for at least 1 s. The same researcher performed all analyses to prevent inter-rater variability [32,33]. Participants were verbally instructed to pull as fast and hard as possible (“ready, go”). Maximum strength and RFD were recorded, with the highest maximum force and mean RFD200 ms used for analysis. Three attempts per hand were performed with 1 min rest between each attempt.

#### 2.3.6. Questionnaires and Scales

During the three measurement sessions and the following days, participants completed a series of questionnaires and scales to monitor various physiological and psychological factors. Sleep duration and quality were recorded on a 1–5 scale, and perceived exertion (RPE) was assessed before and after testing. Mood states, including tension, depression, vigor, fatigue, confusion, and anger, were measured with the Profile of Mood States (POMS) on a 0–4 scale. Participants also reported their beliefs regarding the supplement ingested (caffeine or placebo) before and after testing, as well as any acute or next-day side effects such as headache, sleep disturbances, or increased urination [12].

### 2.4. Statistical Analysis

The a priori sample size calculation indicated that recruiting 16 participants would be sufficient for α = 0.05, 1 − β = 0.8, and an effect size of 0.9, which is similar to values reported in previous studies on other sports disciplines [9]. Thus, 16 individuals were recruited, of whom 13 completed all measurements. Post hoc analyses indicate that, although the small expected changes (<5–10%) could reduce statistical power, the large effect sizes observed in previous studies suggest that the final sample of 13 participants remained adequate to detect meaningful differences.

Data collected in the study were analyzed using the statistical package SPSS v29.0 (SPSS Inc., Chicago, IL, USA). First, the data was assessed for normality using the Shapiro–Wilk test (*p* > 0.05). For normally distributed variables, Student’s *t*-test was used; for non-normally distributed variables, the Wilcoxon test was used. Data were presented as mean ± standard deviation (SD). Statistical significance was set at *p* < 0.05. Effect sizes were expressed as Hedges’s g and interpreted as trivial (0.00–0.19), small (0.20–0.49), medium (0.50–0.79), and large (≥0.80).

## 3. Results

### 3.1. Pull-Up Test

The differences between caffeine and placebo use regarding the pull-up muscular strength test and the pull-up muscular endurance test are shown in Table 2.

#### 3.1.1. Pull-Up One-Repetition Maximum and Muscular Strength Power Test

The effects of caffeine on muscular strength and power during pull-ups at various percentages of one-repetition maximum (1RM) were mixed. At lighter loads (60% 1RM), no meaningful differences were observed between the caffeine (CAF) and placebo (PLA) conditions for mean velocity (V_mean_: 0.727 vs. 0.725 m/s), peak velocity (V_peak_: 1.13 vs. 1.15 m/s), mean power (W_mean_: 504 vs. 507 W), and peak power (W_peak_: 921 vs. 942 W). All corresponding *p*-values were non-significant (*p* > 0.05) and effect sizes (g) were trivial to small (g < 0.1).

At moderate loads (80% 1RM), small improvements were noted in the CAF condition. Participants demonstrated increases in V_peak_ (0.841 vs. 0.805 m/s; +4.5%), W_mean_ (437 vs. 422 W; +3.6%), and W_peak_ (744 vs. 689 W; +8.0%). However, these changes were not statistically significant (*p* > 0.05), with effect sizes ranging from small to moderate (g = 0.158 to 0.348).

At near-maximal and maximal loads of 95% 1RM, CAF showed a 3.1–3.2% increase in V_mean_ (0.302 vs. 0.292 m/s) and W_mean_ (284 vs. 275 W), but neither was statistically significant (*p* > 0.500 and g = 0.204–0.159). While at 100% 1RM, CAF led to a 6.1% increase in V_mean_ (0.226 vs. 0.213 m/s) and a 6.0% increase in W_mean_ (227 vs. 214 W), though these were not statistically significant (*p* = 0.178 and *p* = 0.157, respectively). Across all these heavy-load variables, no comparisons reached statistical significance, and effect sizes were predominantly small.

#### 3.1.2. Pull-Up Muscular Endurance Test

CAF supplementation did not elicit a statistically significant improvement in pull-up muscular endurance. The number of repetitions completed was similar between the CAF and PLA conditions (16.8 ± 7.7 vs. 16.3 ± 7.60 repetitions, *p* = 0.292), representing a non-significant increase of 3.3%. Similarly, no meaningful differences were found for velocity or power metrics during the endurance test. The mean power output was nearly identical (323 W for CAF vs. 324 W for PLA, *p* = 0.926), with a trivial effect size (g = 0.011).

### 3.2. Grip Test

The differences between caffeine and placebo use on grip endurance and maximum strength in dead-hang and RFD are shown in Table 3.

#### 3.2.1. Grip Endurance in Dead-Hang

Caffeine administration decreased grip endurance time during the dead-hang test. The time to exhaustion was lower in the CAF condition compared to PLA (39.8 ± 17.2 s vs. 43.0 ± 17.9 s), representing a −7.4% difference. However, this reduction did not reach statistical significance (*p* = 0.162) and the effect size was small (g = 0.171).

#### 3.2.2. Maximum Grip Strength in Dead-Hang

For maximum grip strength, a small, non-significant increase was observed with caffeine. The maximum weight held was 97.3 ± 19.4 kg in the CAF condition and 95.0 ± 16.4 kg in the PLA condition, representing a 2.4% increase (*p* = 0.060; g = 0.120).

#### 3.2.3. Grip Rate of Force Development (RFD)

Caffeine had no significant effect on the RFD for either the dominant or non-dominant hand. For the dominant hand (DH), RFD was slightly lower with CAF (704 ± 196 N/s vs. 721 ± 230 N/s; −2.2%, *p* = 0.382). A similar, non-significant decrease was observed for the non-dominant hand (NDH) (646 ± 169 N/s vs. 667 ± 150 N/s; −3.2%, *p* = 0.169). The effect sizes for these comparisons were small (g < 0.13). Grip strength (GS) measurements also showed no significant changes, and indicated a trivial effect for the non-dominant hand (0.4% change, g = 0.022).

### 3.3. Questionnaires and Scales

Questionnaire data indicated that participants’ beliefs regarding the ingested supplement were moderately accurate, with 62% correctly guessing caffeine intake. Acute caffeine intake significantly increased the perception of strength/velocity (3.8 ± 0.8 vs. 2.9 ± 0.6; *p* < 0.008, g = 1.20), energy (3.3 ± 0.8 vs. 2.1 ± 0.9; *p* = 0.014, g = 1.32), and alertness (2.5 ± 1.0 vs. 2.0 ± 0.9; *p* = 0.025, g = 0.490) compared to the placebo. No stomach problems (1.1 ± 0.3 vs. 1.3 ± 0.6; *p* = 0.157, g = 0.394), muscle stiffness (2.0 ± 0.8 vs. 2.1 ± 1.0; *p* = 1.000, g = 0.061), irritability (1.4 ± 0.8 vs. 1.7 ± 0.8; *p* = 0.480, g = 0.351), or sleeping difficulties (2.1 ± 1.5 vs. 1.5 ± 0.8; *p* = 0.414, g = 0.460) were found. No differences were observed in the variables relating to mood, RPE scale, or sleep conditions.

## 4. Discussion

This study aimed to investigate the acute effects of a low dose of caffeine (3 mg/kg) on key performance metrics relevant to sport climbing. The primary finding is that caffeine ingestion did not produce a statistically significant or practically meaningful ergogenic effect on pull-up strength, power, endurance, or on grip endurance, maximum strength, and rate of force development (RFD) in a cohort of intermediate-advanced trained male climbers. Contrary to our hypothesis and despite subjective perceptions of increased strength, energy, and alertness, the objective performance data indicate that this acute low-dose caffeine protocol was ineffective as a performance-enhancing supplement for the climbing-specific tasks evaluated.

The results for pull-up performance were characterized by a pattern of mixed, non-significant changes. At 80% of 1RM, we observed modest, non-significant improvements in peak velocity (+4.5%), mean power (+3.6%), and particularly peak power (+8.0%), with effect sizes ranging from small to moderate. This aligns with meta-analytical evidence suggesting that caffeine can enhance muscular power and strength, albeit with considerable variability across studies [12,13]. The trend towards improved power output at this submaximal intensity may suggest a potential for caffeine to enhance performance in dynamic, power-oriented climbing movements, such as dynos or rapid moves between holds. However, the lack of statistical significance (*p* > 0.15 for all 80% 1RM power metrics) and the inconsistent results at other loads preclude any definitive conclusions.

Notably, at both lighter (60% 1RM) and heavier loads (90–100% 1RM), the effects were trivial to small and inconsistent, with some metrics even showing slight decrements under the caffeine condition. This lack of a clear effect on maximal strength is somewhat contradictory to Grgic et al. studies [19,34], which reported caffeine’s ergogenicity for upper-body strength exercises. The disparity could be attributed to the specific demands of the pull-up, a multi-joint exercise in which performance depends on factors beyond pure muscular strength, including central nervous system activation and forearm flexor endurance.

Furthermore, caffeine failed to significantly enhance pull-up muscular endurance, with only a trivial 3.3% increase in repetition number and a negligible effect on power maintenance. This finding contrasts with the well-established ergogenic effect of caffeine on endurance performance in other sports [9,12]. The highly localized fatigue of the forearm flexors during repeated pull-ups, a known limiting factor in climbing [5], may not be sufficiently mitigated by the physiological mechanisms of a 3 mg/kg caffeine dose.

The grip tests revealed one of the more unexpected findings, a non-significant 7.4% decrease in dead-hang endurance time with caffeine. While not statistically significant, this trend is noteworthy and contradicts studies that have reported improved grip endurance with caffeine [19]. The underlying mechanism for this potential negative effect is unclear but could be related to the specific neuromuscular control and pain tolerance required for a sustained, maximal isometric contraction on a small edge. Caffeine-induced alterations in tremor or fine motor control [35], though not measured, could theoretically compromise efficiency during a prolonged dead-hang.

For maximum isometric grip strength, our results showed a small (2.4%) increase that approached statistical significance (*p* = 0.060). This finding is partially consistent with research indicating that caffeine can enhance maximal voluntary contraction force [13,19]. However, the effect size was small (g = 0.120), and when considered alongside the lack of effect on RFD and the negative trend in endurance, this suggests that any potential benefit to peak grip strength is likely minimal and may not translate into a meaningful performance advantage in a climbing context.

The lack of effect on RFD is another critical result. RFD is a crucial determinant of rapid, powerful movements in climbing as well as in static or unbalanced positions, where generating force quickly on very small holds is essential to maintaining grip and avoiding falls [6,21]. Our data indicate that caffeine did not improve the ability to generate force quickly in the finger flexors. This null finding aligns with some [36], though not all [21], previous research on caffeine and RFD, and it may indicate that the dose used was insufficient to enhance neural drive to the specific musculature involved in grip or any dynamic contraction [37].

A striking divergence in our data is the disconnect between subjective perceptions and objective performance. Participants reported significantly greater feelings of strength, energy, and alertness after caffeine ingestion, which is consistent with caffeine’s known adenosine receptor antagonism in the central nervous system [12,13]. This suggests that the caffeine dose was pharmacologically active and sufficient to alter psycho-physiological state. However, these positive perceptions did not translate into improved physical performance. This phenomenon has been observed in other sports [38] and highlights that in climbing, where technical skill and precise movement are paramount, a subjective feeling of increased power may not directly correlate with the ability to execute specific, high-skill tasks like a 1RM pull-up or a maximal dead-hang. It is possible that the low dose used, while sufficient to alter mood, was insufficient to elicit significant peripheral ergogenic effects.

Despite the insights provided, this study has several limitations. The use of force sensors for grip strength assessment is not widespread, and participants were likely unfamiliar with the technique, especially climbers without prior dead-hang-based grip training. This can potentially lead to performance improvements solely due to practice, which could influence our results despite randomization and a familiarization session conducted. Additionally, the small sample size involving participants with different climbing performance levels may also influence certain tests, such as finger flexor strength and endurance, particularly for advanced climbers, where factors like skin condition may have impacted the outcomes. The limited sample size also raises the potential for Type II error, as several variables showed moderate effects that may not have reached statistical significance despite being physiologically relevant. Therefore, non-significant findings should be interpreted with caution. Moreover, pull-up measurements using a linear encoder also presented challenges due to the exercise’s non-linear movement, requiring adherence to a standardized protocol. Ultimately, further studies are needed to establish a consensus on the optimal methods for assessing climbing-specific performance, thereby facilitating future research in this sport discipline.

Finally, several methodological aspects must be considered when interpreting these results. First, the caffeine dose of 3 mg/kg is at the lower end of the ergogenic range typically studied (3–6 mg/kg) [12]. This dose was chosen to minimize side effects and reflect a potential ‘real-world’ low intake; however, it may have been insufficient to produce significant performance changes in a trained cohort. Second, our sample consisted of low-to-moderate caffeine consumers. While this reduces the potential impact of tolerance, a higher dose may be required to elicit ergogenic effects in climbers accustomed to the sport’s unique demands. Third, the laboratory-based nature of the tests, while highly controlled, may not fully capture the complex, intermittent, and skill-dependent nature of actual sport climbing performance on a route. Future research should investigate the effects of different caffeine doses (e.g., 5–6 mg/kg) on sport-specific performance, such as on a climbing-specific treadmill or during redpoint attempts on standardized boulders, and should include female climbers to enhance the generalizability of the findings.

## 5. Conclusions

In conclusion, a low dose of caffeine (3 mg/kg) did not significantly enhance pull-up or grip performance in thirteen intermediate–advanced male climbers. Despite inducing positive subjective states of energy and alertness, the objective metrics of strength, power, endurance, and rate of force development remained largely unchanged, with a notable non-significant trend towards reduced grip endurance.

From a practical standpoint, climbers and their coaches or nutritionists should not expect improvements in strength or endurance from a 3 mg/kg caffeine dose. Its main benefit appears to be cognitive, enhancing focus rather than physical performance, and recommendations should therefore be made with caution. Climbers seeking strength or endurance improvements might require higher doses (e.g., 6 mg/kg), which would need to be monitored carefully to avoid potential adverse effects, especially when training later in the day. They should also consider that individual responses to caffeine can vary, meaning that the effects may differ among climbers. Future studies are warranted to explore the effects of higher doses in larger and more diverse samples of climbers, including women, and to analyze the impact of this supplement using more ecologically valid climbing performance tests.

## Figures and Tables

**Table 1:** Participants’ characteristics.

N	13		
Age (years)	28.2	±	8.60
***Body composition***			
Body mass (kg)	67.1	±	8.30
Fat mass (kg)	6.50	±	3.20
Fat-free mass (kg)	60.6	±	6.20
***Dietary habits***			
Energy intake (kcal/day)	1710	±	493
Protein (g/kg/day)	1.23	±	0.39
Carbohydrate (g/kg/day)	2.76	±	0.99
Fat (g/kg/day)	1.02	±	0.38
***Physical activity and training habits***			
METs (MET-min/week)	10,282	±	1438
Sedentary time (h/day)	7.95	±	2.53
Training sessions (days/week)	2.77	±	0.93
Duration of training sessions (min/session)	133	±	43.2
Training time dedicated to bouldering (min/week)	91.9	±	65.4
Training time dedicated to climbing gym (min/week)	238	±	187
***Athletic performance in climbing***			
Best redpoint grade *	17	±	2.82
Climbing experience (years)	6.25	±	4.29
Self-identified discipline (number of boulderers)	6	±	1
Preferred climbing style (on-sight, n)	7 (54%)		
Preferred terrain (vertical, n)	8 (61%)		
Regular competition participants (n)	3 (23%)		

* According to the classification of Draper et al. [17], the participants had an intermediate-advanced level.

**Table 2:** Differences between caffeine and placebo regarding pull-up muscular strength, power, and endurance tests.

		PLA			CAF			CAF-PLA					
		*Mean*	±	*SD*	*Mean*	±	*SD*	*Mean*	±	*SD*	*p*	g	95% CI
***Muscular strength and power***													
60% 1RM	V_mean_ (m/s)	0.725	±	0.125	0.727	±	0.164	0.002	±	0.089	0.922	0.013	−0.192–0.196
	V_peak_ (m/s)	1.15	±	0.21	1.13	±	0.22	−0.01	±	0.151	0.741	0.086	−0.343–0.315
	W_mean_ (W)	507	±	119	504	±	140	−2.98	±	85.0	0.901	0.022	−188–182
	W_peak_ (W)	942	±	284	921	±	334	−21.2	±	214	0.728	0.063	−487–445
80% 1RM	V_mean_ (m/s)	0.526	±	0.045	0.537	±	0.078	0.011	±	0.063	0.531	0.162	−0.126–0.148
	V_peak_ (m/s)	0.805	±	0.093	0.841	±	0.110	0.036	±	0.123	0.313	0.331	−0.232–0.304
	W_mean_ (W)	422	±	79.0	437	±	98.1	15.2	±	63.5	0.405	0.158	−123–154
	W_peak_ (W)	689	±	129	744	±	165	55.2	±	132	0.157	0.348	−232–343
90% 1RM	V_mean_ (m/s)	0.380	±	0.046	0.388	±	0.053	0.008	±	0.039	0.503	0.151	−0.077–0.093
	V_peak_ (m/s)	0.578	±	0.074	0.574	±	0.078	−0.01	±	0.061	0.832	0.049	−0.137–0.129
	W_mean_ (W)	339	±	59.8	346	±	60.0	6.79	±	34.1	0.486	0.109	−67.5–81.1
	W_peak_ (W)	541	±	115	531	±	99.9	−9.73	±	64.2	0.595	0.131	−150–130
95% 1RM	V_mean_ (m/s)	0.292	±	0.039	0.302	±	0.052	0.010	±	0.054	0.520	0.204	−0.128–0.108
	V_peak_ (m/s)	0.459	±	0.067	0.464	±	0.073	0.004	±	0.077	0.849	0.067	−0.164–0.172
	W_mean_ (W)	275	±	52.2	284	±	53.7	10.1	±	52.0	0.513	0.159	−123–103
	W_peak_ (W)	447	±	96.7	454	±	73.3	4.56	±	85.1	0.856	0.076	−181–190
100% 1RM	V_mean_ (m/s)	0.213	±	0.043	0.226	±	0.053	0.013	±	0.049	0.178	0.252	−0.094–0.120
	V_peak_ (m/s)	0.374	±	0.063	0.368	±	0.099	−0.01	±	0.082	0.394	0.068	−0.185–0.173
	W_mean_ (W)	214	±	61.5	227	±	67.8	12.9	±	44.2	0.157	0.188	−83.4–109
	W_peak_ (W)	394	±	134	389	±	164	−4.88	±	93.9	0.427	0.031	−209–200
***Muscular endurance***													
Rep (n)		16.3	±	7.60	16.8	±	7.68	0.538	±	1.76	0.292	0.061	−3.30–4.37
V_mean_ (m/s)		0.484	±	0.104	0.487	±	0.116	0.003	±	0.051	0.813	0.025	−0.108–0.114
V_peak_ (m/s)		0.773	±	0.169	0.782	±	0.189	0.008	±	0.073	0.688	0.047	−0.151–0.167
W_mean_ (W)		324	±	90.1	323	±	82.1	−0.96	±	36.8	0.926	0.011	−81.1–79.2
W_peak_ (W)		600	±	231	591	±	204	8.67	±	109	0.779	0.040	−229–246

Data is presented as mean ± SD. Abbreviation: 1RM, one-repetition maximum; CAF, caffeine; PLA, placebo; V_mean_, mean velocity; V_peak_, peak velocity; W_mean_, mean power; W_peak_, peak power.

**Table 3:** Differences between caffeine and placebo in grip strength tests.

	PLA			CAF			CAF-PLA					
	*Mean*	±	*SD*	*Mean*	±	*SD*	*Mean*	±	*SD*	*p*	g	95% CI
***Grip endurance in dead-hang***												
Time (s)	43.0	±	17.9	39.8	±	17.2	−3.18	±	11.1	0.162	0.171	−27.4–21.0
***Maximum GS in dead-hang***												
Weight (kg)	95.0	±	16.4	97.3	±	19.4	2.31	±	4.97	0.060	0.120	−8.52–13.1
***Grip rate of force development***												
GS DH (N)	417	±	93	431	±	100	14.1	±	4.1	0.120	0.136	−75.4–104
GS NDH (N)	403	±	76	405	±	92	1.49	±	3.4	0.439	0.022	−72.8–75.8
RFD DH (N/s)	721	±	230	704	±	196	−16.2	±	3.8	0.382	0.059	−855–823
RFD NDH (N/s)	667	±	150	646	±	169	−21.5	±	7.8	0.169	0.123	−191–148

Data is presented as mean ± SD. Abbreviation: DH, dominant hand; CAF, caffeine; GS, grip strength; NDH; Non-dominant hand; PLA, placebo; RFD, rate of force development.

## Data Availability

The original contributions presented in the study are included in the article; further inquiries can be directed to the corresponding author.
